# Joint analyses of open comments and quantitative data: Added value in a job satisfaction survey of hospital professionals

**DOI:** 10.1371/journal.pone.0173950

**Published:** 2017-03-15

**Authors:** Ingrid Gilles, Mauro Mayer, Nelly Courvoisier, Isabelle Peytremann-Bridevaux

**Affiliations:** University Institute of Social and Preventive Medicine (IUMSP), Lausanne University Hospital, Lausanne, Switzerland; Public Library of Science, FRANCE

## Abstract

**Objective:**

To obtain a comprehensive understanding of the job opinions of hospital professionals by conducting qualitative analyses of the open comments included in a job satisfaction survey and combining these results with the quantitative results.

**Design:**

A cross-sectional survey targeting all Lausanne University Hospital professionals was performed in the fall of 2013.

**Material and methods:**

The survey considered ten job satisfaction dimensions (e.g. self-fulfilment, workload, management, work-related burnout, organisational commitment, intent to stay) and included an open comment section. Computer-assisted qualitative analyses were conducted on these comments. Satisfaction rates on the included dimensions and professional groups were entered as predictive variables in the qualitative analyses.

**Participants:**

Of 10 838 hospital professionals, 4978 participated in the survey and 1067 provided open comments. Data from 1045 respondents with usable comments constituted the analytic sample (133 physicians, 393 nurses, 135 laboratory technicians, 247 administrative staff, including researchers, 67 logistic staff, 44 psycho-social workers, and 26 unspecified).

**Results:**

Almost a third of the comments addressed scheduling issues, mostly related to problems and exhaustion linked to shifts, work-life balance, and difficulties with colleagues’ absences and the consequences for quality of care and patient safety. The other two-thirds related to classic themes included in job satisfaction surveys. Although some comments were provided equally by all professional groups, others were group specific: work and hierarchy pressures for physicians, healthcare quality and patient safety for nurses, skill recognition for administrative staff. Overall, respondents’ comments were consistent with their job satisfaction ratings.

**Conclusion:**

Open comment analysis provides a comprehensive understanding of hospital professionals’ job experiences, allowing better consideration of quality initiatives that match the needs of professionals with reality.

## Introduction

Job satisfaction has been extensively studied for more than 50 years, with approximately one third of publications dedicated to hospital professions. Interest in it is currently re-emerging as a concern for hospital governance. Indeed, during the past decades, several ‘transitions in health care’[1] have marked hospital functioning:[2] population ageing, an increase in the prevalence of chronic diseases and multi-morbidity, cost-reduction policies, and a predicted shortage of healthcare professionals.[3, 4] These transitions have led to important changes in managerial practices and, ultimately, in professionals’ working conditions.[2] As a result of time pressures and the introduction of skill mixes, tasks have become more technical and professionals more interdependent. This is characterised by more complex decision making processes, and potential inter-professional role conflicts leading to hardship at work and high turnover.[5–8] In such contexts, job satisfaction should be carefully considered by hospital administrators, especially as scholars have pointed out its influence not only on professionals’ health,[9, 10] but also on patients’ satisfaction,[11, 12] quality of care, and safety.[13–17]

Job satisfaction is considered to be an attitude.[18, 19] As such, it is defined as ‘the sum of the evaluations of the discrete elements of which the job is composed’.[20] In other words, it is viewed as the subjective rating of different aspects that contribute to people’s work situation or experiences.[18, 21] It is thus usually assessed by self-reported questionnaires that measure job dimensions.[22, 23] Many studies have now reported evidence of the strength of this construct. However, as a self-reported measure, job satisfaction is still perceived as being liable to potential bias such as social desirability or acquiescence.[24, 25] Moreover, the choice of dimensions included in questionnaires is not exhaustive, because the knowledge of a job situation is restricted to selected dimensions representing only a small part of the entire work context.[26, 27] This can be a real issue in hospitals, which are characterised by a large heterogeneity of professional situations.

Taking advantage of open comments generally included at the end of satisfaction questionnaires would represent a complementary solution to the classic use of self-reported measures of job satisfaction.[28] According to Stoneman *et al*,[28] open comments allow direct access both to the ‘frame of reference’ of professionals, which can significantly differ from those of the questionnaires designers, and to ‘more heterogeneous sets of perspectives than the standard closed-format question’. Despite these advantages, open comments have rarely been analysed as proper qualitative material; they have been used even less for mixed analyses combining both open comment results and the quantitative results of the questionnaires in which they are included.[28] Linking quantitative results with a qualitative analysis of open comments would provide (1) a more comprehensive understanding of work aspects or situations that hospital professionals relate to satisfaction or dissatisfaction, (2) information on the comprehensiveness of job satisfaction dimensions included in the questionnaire, and (3) indications about the consistency between the quantitative evaluations and the discourse produced about job situations (indirect check of desirability bias). The aim of this study was to address these three objectives.

## Material and methods

### Setting

Lausanne University Hospital is one of five Swiss university hospitals. It is a tertiary healthcare centre of approximately 1500 beds comprising acute care departments, geriatric rehabilitation, psychiatric wards, and a long-term care facility in separate buildings. It employs approximately 11 000 professionals whose characteristics do not differ from those found in other university hospitals in Switzerland: two thirds are women, half are employees over 40 years old, and half are professionals who work with patients.

### Sample and data collection

The Lausanne University Hospital cross-sectional job satisfaction survey has been conducted every 2 years since 2007. In the present study, we used data from the 2013 survey (collection give up between September 17, 2013, and November 4, 2013), which targeted all 10 838 hospital employees (see Respondents’ characteristics for occupations considered). They were contacted by e-mail and by mail, and they could respond by using an electronic or a paper version of the questionnaire. They received two electronic reminders. The survey was completely anonymous and respondents were free not to respond or to abandon the survey completion at any time.

### Measures

The Lausanne University Hospital survey (described in detail elsewhere)[7] comprised 34 items allowing the measurement of 10 job satisfaction dimensions and an overall job satisfaction index (S1 Appendix), as well as an open comments section.

### Job satisfaction dimensions

The job satisfaction dimensions included in the questionnaire were as follows: manager characteristics (seven items; α = 0.74), workload (three items; α = 0.64), career opportunities (two items; r = 0.37), working conditions (five items; α = 0.79), co-worker support (two items; r = 0.56), professional fulfilment (two items; r = 0.42), work-related burnout (seven subscale items from the Copenhagen Burnout Inventory; α = 0.87),[29] organisational commitment (three items; α = 0.64), and intent to stay (one item). Work organisation was also measured but not considered for analysis because of its weak reliability (two items; r = 0.01).

Other than for emotional and work exhaustion, respondents rated whether they were satisfied with each item on a Likert scale ranging from 1 (not at all) to 4 (yes, absolutely). For work-related burnout, respondents rated the frequency of exhausting situations on a Likert scale ranging from 1 (always) to 5 (almost never).

For each dimension other than intent to stay, mean scores were computed. This calculation allowed a percentile-based categorisation of respondents into three levels of satisfaction (or frequency for emotional and work exhaustion): high, medium, and low. For intent to stay, we created two categories of respondents, which differentiated those who intended to stay working at the hospital from those who intended to quit over the coming years.

### Overall job satisfaction

This dimension was measured by a single item:[30] respondents had to rate their general level of professional satisfaction on a scale ranging from 1 (not satisfied) to 10 (extremely satisfied). Like the job satisfaction dimensions, three levels of percentile-based satisfaction were computed for this dimension.

### Open comments

At the end of the questionnaire, respondents could write or type a free comment about their job (instruction: ‘Please give us feedback or comments about your job’). The open comments section was one page long in the paper questionnaire and of unlimited length in the electronic version.

### Personal information

The following information was asked of the respondents and considered in the analyses: their hierarchical level (managers vs. non-managers), their professional group (physicians, care providers, laboratory staff, administrative staff, researchers, logistic staff, and psycho-social workers), and their department (medicine, surgery, medico-surgical facilities, psychiatry, laboratory and radiology, administration, hospital logistics, and research). Gender, age, work contract type and years of working in the hospital were also asked to respondents but as these variables had no effects, they were removed from analyses. Respondents were free to provide or not this personal information.

### Analytical strategies

#### Sample checks

We conducted a chi-square analysis to ensure that respondents’ characteristics (sex, age group, years of working in the hospital, hierarchical level, professional group, department, etc) did not differ among the overall hospital employees, the respondents’ sample, and the respondents who left a comment. Independent samples t-tests were used to compare the job satisfaction ratings of respondents who left a comment or did not. Because of the large sample size, an effect-size indicator (Cohen’s d) served as the significance estimator (d ≈ .20 small; d ≈ .50 moderate; d ≈ .80 strong) for this test.

#### Computer-assisted qualitative analyses

All open comments were compiled to form an overall text. They were then analysed with the software IRaMuTeQ (IRaMuTeQ; 2008–2014 Pierre Ratinaud) by using the Reinert method.[31–32] Both the software and the method are particularly appropriate for the analyses of large texts composed of short sentences that make manual coding difficult; they are thus recommended for analyses of open comments.[28] According to this method, the software first establishes a list of the vocabulary in the entire text, reducing words to their roots, on the basis of pre-established dictionaries (eg, *nurse* and *nursing* reduced to a common entity *nurs+*). It then constructs different patterns of vocabulary distribution in order to identify discourse classes; these patterns are obtained by using automatic iterative descending hierarchical classifications to the analysed text. In other words, on the basis of their co-occurrences, pairs of words and sentences that are statistically frequently associated are gathered into the same class of discourse, and words that are less frequently associated form distinct classes. Chi-square tests provide a statistical indication of the strength of the association between vocabulary and classes: for a given class, words or excerpts that are statistically over-represented are referred to as *typical*, whereas those that are statistically under-represented (but relevant for other classes) are referred to as *anti-typical*. It is then up to the researcher to label the classes according to his or her interpretation of typical or anti-typical words or excerpts.

By computing the χ^2^ test, the software estimated the strength of associations between classes of discourse and modalities of the following variables, extracted from the quantitative part of the survey: overall job satisfaction, job satisfaction dimensions, hierarchical level, professional group, and department. This allowed us to know whether some classes of discourse were significantly present or absent from the comments of specific respondents, as characterised by these variables (their level of satisfaction on dimensions, their professional groups, their department, etc).

As we had more than 1000 comments, and because of the heterogeneity of topics usually found in open comments,[28] we opted for a two-step strategy for our analysis. In the first step, we considered the entire text as the analytic material, and we considered the main classes of discourse structuring the comments. In the second step, we had the software extract pieces of text that were representative of each main class of discourse identified in the first step. We then considered these pieces of text as separate analytic materials, which were used for further analyses and to obtain more discrete classes of discourse.

## Results

### Respondents’ characteristics

The characteristics of respondents are reported in Table 1. Of the 4978 respondents (response rate of 45.9%), 1067 (21.4%) made an open comment. Twenty-two comments were removed because they were inappropriate for analyses (eg, ‘Nothing to report’; ‘Nothing’; indication of date; respondent’s name and surname). The analytical sample thus included comments from 1045 respondents whose characteristics were similar to those of all survey respondents and to those of all hospital employees.

**Table 1:** Hospital employees and survey respondents’ characteristics.

	Hospital employees (%)	Survey respondents (%)	Survey respondents who wrote a comment (%)
**N**	10 838	4978	1045
**Sex**			
Men	31.1	31.8	28.8
Women	68.9	67.4	70.4
Missing	—	0.8	.08
**Age, years**			
< 30	18.6	18.5	15.8
30–39	32.1	31.4	30.9
40–49	24.4	24.6	25.8
≥ 50	24.9	24.6	27.1
Missing	—	0.9	0.5
**Years of working in the hospital**			
< 3	32.7	20.1	19.3
3 to 5	23.0	22.9	21.0
6 to 10	18.1	20.3	21.0
> 10	26.2	35.7	37.7
Missing	—	1.0	1.0
**Organisational status**			
Managers	9.3	11.4	10.8
Non-managers	90.7	88.6	89.2
**Occupation**			
Physicians	15.8	12.4	12.7
Nurses and care providers	35.7	33.9	37.6
Laboratory staff	10.5	12.5	12.9
Administrative staff	16.6	20.4	18.5
Researchers (excl. physicians)	3.0	5.4	5.2
Logistic staff	10.3	7.8	6.4
Psycho-social workers	3.4	3.9	4.2
Other	4.8	2.5	2.0
Missing	—	1.1	0.5
**Facilities**			
Medicine	14.7	13.7	15.1
Surgery	17.3	15.3	16.6
Medico-surgical	21.0	19.2	20.4
Psychiatry	15.2	13.9	13.4
Laboratory and radiology	8.0	9.1	10.2
Administration	10.1	10.9	9.7
Logistics	9.2	6.1	5.2
Research	4.4	4.9	5.0
Missing	—	6.9	4.4

The only noticeable difference concerned the logistic staff, which was under-represented (10.3% of hospital employees and 6.4% of the analytic sample).

Comparisons of levels of overall satisfaction and of satisfaction on dimensions between respondents who gave and did not give a comment are presented in Table 2. They show that respondents who provided a comment were slightly less satisfied than those who did not provide a comment. This was the case in all dimensions except for work environment and organisational commitment. However, effect sizes were small for a majority of dimensions and moderate for overall job satisfaction and work-related burnout. This finding suggests that there are no relevant differences in satisfaction between respondents who made a comment and those who did not.

**Table 2:** Global and dimension job satisfaction level (mean scores and standard deviations) as a function of the presence vs. absence of a comment at the end of the survey.

	Respondents who did not propose a comment (n = 3914)		Respondents who proposed a comment (n = 1045)		
	M	SD	M	SD	Cohen’s d
Overall job satisfaction	6.9	1.7	6.1	2.1	.42
Self-fulfilment	3.2	0.6	3.1	0.6	.17
Supervisor characteristics	3.1	0.5	3.0	0.6	.18
Co-worker support	3.2	0.6	3.1	0.6	.17
Emotional and work exhaustion	3.3	0.7	3.0	0.8	.40
Career opportunities	2.3	0.5	2.5	0.8	.30
Workload	2.9	0.6	2.6	0.7	.46
Work environment	3.1	0.6	3.1	0.6	.00
Organisational commitment	3.2	0.5	3.2	0.5	.00
Intent to stay	3.3	0.7	3.1	0.8	.27

Scores for overall job satisfaction ranged from 1 (not satisfied at all) to 10 (extremely satisfied); scores for work-related burnout ranged from 1 (always) to 5 (almost never); scores for remaining dimensions ranged from 1 (not satisfied at all) to 4 (totally satisfied). All differences are statistically significant because of the large sample size. So that this issue can be managed and meaningful differences identified, the effect size (Cohen’s d) is reported in the table instead of t-test and p-values (d ≈ .20 small; d ≈ .50 moderate; d ≈ .80 strong).

### Comments analysis

Our two-step analysis consisted of (1) an analysis of the entire text to identify the main discourse classes and (2) analyses of pieces of text corresponding to these main classes to identify the subclasses that they are composed of. The lexicometric characteristics of the texts analysed are presented in S2 Appendix.

#### Analysis of main discourse classes (step 1)

**Fig 1:**
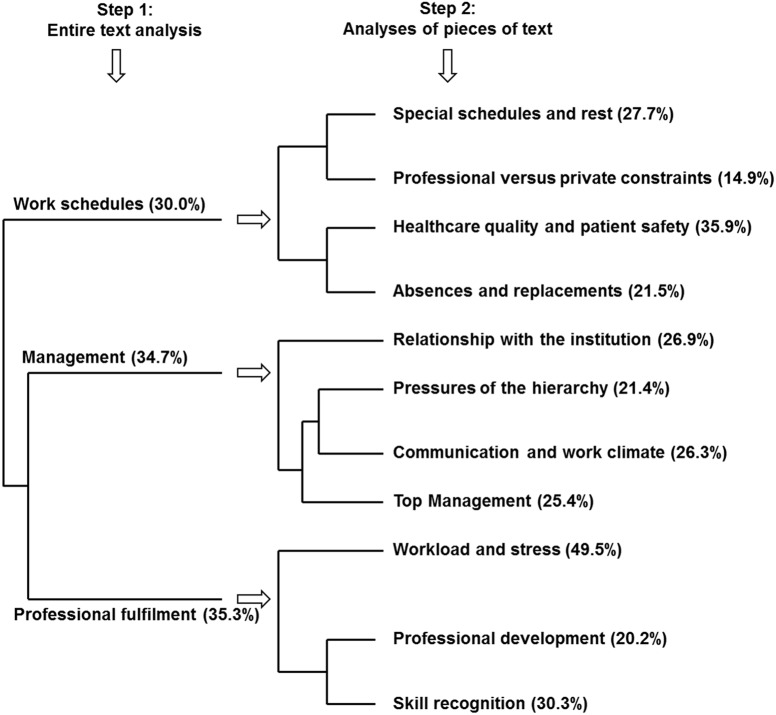
Dendrogram representing the main classes and subclasses resulting from the IRaMuTeQ descending hierarchical classification analysis of the corpus. Main class and subclass labels show the percentage of classified text segments.

We analysed a total of 76 471 words. Among these words, 2712 were repeated 16 times on average. This first result, alongside a weak index of vocabulary richness, indicated redundant discourses. Three distinct main discourse classes, which included 90.2% of the entire text, emerged from the descending hierarchical classification (Fig 1). Chi-square analysis associated with typical words, anti-typical words, or excerpts of each class revealed that the first main class included respondents’ discourse about work schedules (30.0% of the analysed text); the second discourse about management (34.7% of the analysed corpus), and the third discourse about professional fulfilment (35.3% of the analysed corpus). The descending hierarchical classification (Fig 1) also indicated that discourse about management was more often given in relation to aspects of professional fulfilment than to work schedule issues.

### Analyses of subclasses and quantitative data correspondence (step 2)

In step 2, analyses of the subclasses extracted from the three main classes that emerged from step 1 provided information on specific themes approached by respondents in each main class. Table 3 summarises the most typical and anti-typical words or excerpts from subclasses corresponding to each main class. Chi-square analyses of the correspondence between subclasses and categories of respondents or dimensions are reported in S3 Appendix.

**Table 3:** Most typical words, anti-typical words, and typical excerpts from each class and subclass arising from the open comment analyses (ranked in order of importance).

Main classes and subclasses	Typical words	Anti-typical words	Typical excerpt
**Work schedules**			
Special schedules and rest	Day, weekend, timetable, night, week, hour, public holiday	Patient, place, to take, staff, collaborator, material, to replace	• I have irregular hours; I work during evenings, weekends, and public holidays. • The irregular rhythm, days, nights are exhausting in the end.
Professional versus private constraints	Place, CHUV, child, nursery, parking, Lausanne, scandalous	Patient, work, night, day, holiday, to take, condition, to replace	• Lack of places in the nursery is a critical problem. • There are not enough places in the nursery to retain competent professionals in the services.
Healthcare quality and patient safety	Patient, bed, to take, meal, restaurant, security, quality	Timetable, holiday, day, hour, illness, to replace, to pay	• Working in too small premises […] is incompatible with patients care who talk about private things. • By removing some working hours, there is less security for patient care.
Absences and replacements	To replace, illness, motherhood, mistake, colleague, woman, frustration	Timetable, place, day, hour, CHUV, child, bed	• Women coming back from maternity leave are transferred into other services without acknowledging it. • Not enough staff, professionals not replaced, workload increased.
**Management**			
Relationship with the institution	To work, CHUV, pleasure, part, to come, happy, condition	Head, unit, responsible, superior, collaborator, department, time	• I like to work with you; the team is very nice and pleasant. • I am very proud to work at CHUV.
Pressures of the hierarchy	Pressure, head, to listen, manager, training, decision, colleague	CHUV, to work, within, hierarchical, part, responsible, motivation	• Very bad support from hierarchy in particular. • I would like managers to treat all collaborators in the same way.
Communication and work climate	Responsible for, hierarchical, communication, difficult, within, ambiance, direct	To work, to see, CHUV, direction, human resources, health	• The lack of communication between departments, teams, services, colleagues and also managers is blatant. • In every team, some elements spoil ambiance.
Top management	Direction, care, director, account, human resources, to hold, to become	To work, team, to feel, difficult, report, to support, collaboration	• […] small unit with little management. The direction is distant from what happens concretely. • […] By chance my manager is a human care director, attentive and fair.
**Professional fulfilment**			
Workload and stress	To be in charge of, time, work, patient, to increase, team, administrative	To work, to see, CHUV, direction, human resources, health	• Workload has considerably increased these last years. • Workload does not stop growing and is more and more associated with administrative tasks.
Professional development	Training, to offer, course, superior, field, position, promotion	CHUV, to work, inside, hierarchical, part, responsible, motivation	• My activity is satisfying; however, the training possibilities are too limited. • The possibility to stay informed of the state of science and knowledge by participating in congresses should be increased or stimulated.
Skill recognition	Skill, professional, to recognise, CHUV, to like, class, salary	Head, unit, responsible, superior, collaborator, department, time	• Interesting activity but my skills are not considered. • I would like to use my professional skills more.

All words in the table are significantly (p<0.05) linked with classes or subclasses on the basis of Chi squares with 1 df. CHUV = Lausanne University Hospital (in French: Centre Hospitalier Universitaire Vaudois).

***Subclasses corresponding to the ‘work schedules’ main class*:** The analysis of the text relative to work schedules highlighted four subclasses. The first, consisting of 27.7% of the classified text, concerned special schedules and rest. In this subclass, respondents commented on the hardship of some working schedules, such as night or weekend work, and the recovery system that was mostly judged as inappropriate. This subclass was essentially composed of concrete examples and of numbers of working hours. Chi-square analyses indicated that laboratory workers and physicians, as well as respondents who were poorly satisfied with their career opportunities and willing to leave the hospital in the coming years but not experiencing work-related burnout, were significantly over-represented in this subclass. On the other hand, nurses, professionals working in surgical facilities, and respondents who were strongly committed to the hospital or who had a strong intent to stay raised this topic less often than other topics related to work schedules.

The second subclass comprised 14.9% of the classified text and concerned professional versus private constraints. Respondents referred to difficulties in duly managing work-life balance, mainly issues related to children: day nurseries, compatibility between family life and full-time work, etc. Although this subclass was over-represented in the discourse of respondents working in the logistics department, who were satisfied with their career, who were committed to the hospital, or who had moderate work-related burnout, it was absent from the discourse of nurses, employees working in medico-surgical facilities, and those who had work-related burnout.

The third theme comprised 35.9% of the classified text and concerned healthcare quality and patient safety. More precisely, respondents pointed out elements that, to their mind, decreased the quality and safety of patient care. These elements were heterogeneous, including issues such as communication between facilities, understaffing, patients’ cultural differences, equipment, and workload. Nurses’ comments were over-represented in this theme, as well as those of respondents with a strong intent to stay at the hospital and those who were moderately satisfied with their professional fulfilment or with their career. Antithetically, laboratory staff, or more surprisingly, physicians, commented about this theme less often than they did about other themes, as did respondents who were highly fulfilled with their work or unsatisfied with their career.

The last subclass relating to work schedules included 21.5% of the classified text and tackled the challenge of management of absences and replacements. It mainly concerned occupational risks, health concerns, maternity leave, and replacement by on-call temporary staff. Nurses, professionals working in medical and surgical facilities, respondents with work-related burnout, or those who were unsatisfied with their workload mostly raised this topic, whereas respondents from laboratory and radiology departments and those who did not have work-related burnout did not. It is worth noting that when respondents commented on absences and replacements, they tended to talk also about healthcare quality and safety, but not about special schedules or private/professional constraints and vice versa (cf. Fig 1).

***Subclasses corresponding to the ‘management’ main class*:** The analysis of the text relative to management highlighted four subclasses. The first involved 26.9% of the classified text and was distinct from the other three (cf. Fig 1). In this subclass, respondents expressed their relationship with the institution (overuse of ‘I’ and ‘me’) and, more precisely, their commitment to the hospital. This discourse was characteristic of non-managers, administrative staff, and employees working in research/education facilities, or of respondents who felt committed or willing to stay working at the hospital and who were globally satisfied with their workload, their managers, and their fulfilment. It was less characteristic of physicians, managers, or respondents who were dissatisfied with their career, the way they were managed, and their fulfilment at work.

The second subclass involved 21.4% of the classified text and targeted pressures of the hierarchy. Topics raised concerned mainly the support or lack of empathy from immediate supervisors, as well as the context, time, or hierarchical (not only from direct managers) pressures. Physicians and respondents who were dissatisfied with their career were over-represented in this subclass, whereas administrative staff and respondents who were moderately satisfied with their career were under-represented.

The third subclass comprised 26.3% of the classified text and focused on communication and work climate. Respondents drew an automatic link between communication issues and climate in teamwork. Comments from psycho-social workers, researchers, or respondents committed to the hospital were typically in line with this subclass. In terms of the respondents’ discourse, this subclass was close to that in the hierarchy pressures subclass.

The fourth subclass consisted of 25.4% of the classified text and related to top management in general. Respondents discussed strategic orientations and their disconnection from the team, both at hospital and at department levels. Surprisingly, the discourse of managers was over-represented. It was also present in respondents who wanted to leave their current position, who were globally dissatisfied, or who raised professional fulfilment concerns. Non-managers, committed respondents, or those who were satisfied with their workload and willing to stay at their position did not generate this type of comment.

***Subclasses corresponding to the ‘professional fulfilment’ main class*:** The analysis of the text relative to management highlighted three subclasses. The first involved almost half of the classified text (49.5%) and was distinct from the other two. It concerned workload and stress. Respondents noted an increase in workload, essentially because of the number and severity of cases, as well as how cumbersome the administrative tasks were. They also pointed out the consequences of this increase in terms of exhaustion and occupational stress. Physicians, respondents working in medical departments, those with work-related burnout, and those who were dissatisfied with their workload or their professional fulfilment were significantly over-represented. In contrast, respondents who were satisfied with their workload, who were moderately fulfilled, or who were dissatisfied with their career did not produce this type of comment.

Comments that were classified as belonging to the second and third subclasses, which addressed professional development and skill recognition, were often made conjointly, and accounted for 20.2% and 30.3% of the classified text, respectively. Regarding professional development, respondents commented on the difficulty they had in acquiring new skills and evolving in the institution because of the mismatch between expected and afforded training, or because of the difficulty they had to attend this training because of time pressure or lack of support from the hierarchy. This subclass was typical of researchers, of respondents working in medico-surgical facilities, of those who were moderately professionally fulfilled, or those who were committed. Regarding skill recognition, respondents commented, in either positive or negative terms, on the recognition of already acquired skills. This was typical of employees, administrative staff, respondents working in psychiatric facilities, and respondents dissatisfied with their career, but not those with work-related burnout or who felt overwhelmed with work. Conversely, the discourse about skill recognition was significantly absent from physicians’ or managers’ comments, as well as from respondents who were not satisfied with their workload or who had work-related burnout.

## Discussion

Our study focused on open comments transmitted by hospital professionals in the context of an institutional job satisfaction survey, and on the added value of joint analyses of open comments and quantitative data. We aimed to identify themes that respondents judged as being relevant to their situations, to consider the logical associations they made between these themes, and to check the congruence between the latter results and the quantitative assessments of job satisfaction. Analyses showed interesting results.

First, we observed that respondents commented on dimensions that are classically discussed in the job satisfaction literature: although some dimensions were included in the questionnaire (workload, professional development, work-life balance, etc), others were not, such as skill recognition and assessment of top management. We also observed that a third of the comments dealt with schedules, suggesting that a larger part of the questionnaire could be dedicated to this theme. Open comment analysis thus appeared to be a complementary and valuable tool in the job satisfaction evaluation process.

Second, logical associations between themes showed that concerns about healthcare quality and patient safety were clearly raised in co-occurrence with colleagues’ absence and less with the exhaustion of work shifts or the lack of rest. This finding suggests that healthcare quality is an institutional rather than an individual concern. Another association showed that, despite the fact that workload and stress were raised in several classes of discourse (schedules, pressures of the hierarchy), they were mainly perceived by respondents as limitations to professional development and skill recognition. In addition, we observed findings in line with the literature about job satisfaction cultures and subcultures in hospital professional groups.[3, 33] In fact, themes were approached differently and had different associations depending on the professional groups. These results can be illustrated, for example, by the fact that physicians were predominantly concerned about pressure at work, and pointed out schedules, hierarchy, and workload as sources of pressure. Although healthcare quality and patient safety were absent from their comments, these were over-represented in nurses’ comments. Other professionals focused on organisational aspects of their work (work/life balance, commitment, communication/work climate, professional development, and skill recognition). These results suggest, therefore, that pressures, patient care, and organisational context are perceived by physicians, nurses, and remaining professionals, respectively, as critical aspects of their job. The absence of association between healthcare quality and job satisfaction in the physicians’ comments could seem surprising, especially in an institutional context of enhancing quality indicators.[34] When we reviewed the literature on physician job satisfaction, the perception of healthcare quality was rarely described as a direct and strong predictor of job satisfaction. In fact, it has been shown to be only indirectly associated with satisfaction through job autonomy[35] or quality of the relationship with patients;[36] when a direct association was observed, it was quite modest.[37]

The third interesting result relates to the links between the level of satisfaction of the dimensions and the discourse transmitted in the open comments. These links showed that themes discussed by respondents were mostly consistent with their level of satisfaction on related dimensions. For example, respondents who were not satisfied with their workload were over-represented in the comments about workload and stress. Similarly, respondents who were unsatisfied with their career perspectives were over-represented in comments about skill recognition, and respondents committed to their institution were over-represented in discourses about their relationship with the institution. This consistency argues in favour of the reliability of the quantitative measure, but also highlights truly actionable options as interventions. For example, respondents who had a strong intent to leave mainly discussed work time planning issues and the gap they felt between these issues and the decisions made by top management. Those who felt they had work-related burnout spoke mainly about irregular schedules, the ineffective system of absence management, and workload.

This study presents several strengths: the use of spontaneous textual data from respondents about their job situations, which adds empirical value to the results; a large sample size for such types of analyses; and the links between the survey’s quantitative results and the respondents’ open comments, allowing a more comprehensive understanding of job satisfaction issues. However, the study also has two limitations to consider. First, the fact that only a fifth of the survey respondents wrote a comment may limit its representativeness. Nonetheless, representativeness is not a goal per se in qualitative analyses, which aim to collect enough material to reach information saturation, which occurred in this study. Second, the results may not be generalisable to other healthcare contexts.

In future research in hospital professionals’ job satisfaction, open comments should be more systematically proposed and analysed to complement quantitative results. There are at least two reasons for this. Firstly, job satisfaction surveys are dedicated to allow respondents’ expression of their opinions about their job, and researchers or deciders to collect knowledge about work context and processes. In such situations, closed-ended questions cannot completely help reaching these two goals because answer modalities cannot be comprehensive in terms of choice of opinions and experiences.[38] Secondly, the variety of hospital work settings [39, 40] cannot be captured by a standardized job satisfaction survey, even if designed for specific professional groups. Open comments are therefore efficient for contextualizing quantitative results from job satisfaction surveys, and for providing concrete suggestions to hospital managers.[41] To summarize, beyond the fact that open comments can confirm or complement quantitative results from job satisfaction questionnaires, they may also offer concrete information about corrective and strategic measures that hospital governance could consider implementing.

## Supporting information

S1 Appendix Dimensions and items included in the quantitative analyses (with Cronbach alpha’s and rating scales). (DOCX)

S2 Appendix Principal lexicometric characteristics of the different texts analysed. (DOCX)

S3 Appendix Signed chi squars indicating significant over- and under-representativeness of independent variable modalities in each thematic class. (DOCX)
