# Supplementary material for: Joint analyses of open comments and quantitative data: Added value in a job satisfaction survey of hospital professionals
Source: PLoS One. 2017 Mar 15;12(3):e0173950. doi: 10.1371/journal.pone.0173950 (PMC5352002; doi:10.1371/journal.pone.0173950)
Supplement: S1 Appendix — (DOCX) [file pone.0173950.s001.docx]

| **S1 Appendix.** Dimensions and items included in the quantitative analyses (with Cronbach alpha’s and rating scales) | | |
| --- | --- | --- |
| Dimensions and items | Rating scales | Cronbach alphas |
| **Manager characteristics** | -- | .74 |
| 1. Your manager is available when you need his/her help | 4 - points |  |
| 2. Your manager appreciates your work | 4 - points |  |
| 3. Your manager recognizes your competences | 4 - points |  |
| 4. Your manager supervises you sufficiently | 4 - points |  |
| 5. Your manager is respectful with his/her team members | 4 - points |  |
| 6. Your manager behaves fairly with all team members | 4 - points |  |
| 7. Your manager leads and motivates his team satisfactorily | 4 - points |  |
| **Workload** | -- | .64 |
| 1. Can you accomplish your work within the scheduled time? | 4 - points |  |
| 2. Do you feel that workload is distributed with equity in your team? | 4 - points |  |
| 3. Does your work situation (e.g. schedules, holidays, leisure) allow work and private life combination? | 4 - points |  |
| **Career opportunities** | -- | .37^a^ |
| 1. Do you think that your professional development is encouraged by your superiors? | 4 - points |  |
| 2. In your opinion, is a career perspective possible for you in the institution (e.g. promotion, mobility)? | 4 - points |  |
| **Working conditions**  Are the following working conditions well suited to your work demands | -- | .79 |
| 1. facilities | 4 - points |  |
| 2. equipment | 4 - points |  |
| 3. hygiene conditions | 4 - points |  |
| 4. security | 4 - points |  |
| 5. maintenance and services | 4 - points |  |
| **Work organization** | -- | .01^a^ |
| 1. Globally, would you say that the work is well organized in your facility? | 4 - points |  |
| 2. Is information sharing well organized in your facility? | 4 - points |  |
| **Co-workers support** | -- | .56^a^ |
| 1. Can you count on your colleagues’ support? | 4 - points |  |
| 2. Do you feel that relationships in your facilities are respectful? | 4 - points |  |
| **Professional fulfillment** | -- | .42^a^ |
| 1. Is your work the occasion to use your skills and abilities? | 4 - points |  |
| 2. Do you enjoy coming to work? | 4 - points |  |
| **Work-related burnout** | -- | .87 |
| 1. Is your work emotionally exhausting? | 5 - points |  |
| 2. Do you feel burnt out because of your work? | 5 - points |  |
| 3. Does your work frustrate you? | 5 - points |  |
| 4. Do you feel worn out at the end of the working day? | 5 - points |  |
| 5. Are you exhausted in the morning at the thought of another day at work? | 5 - points |  |
| 6. Do you feel that every working hour is tiring for you? | 5 - points |  |
| 7. Do you have enough energy for family and friends during leisure time? (reversed) | 5 - points |  |
| **Organisational commitment** | -- | .64 |
| 1. Are you proud to work within this hospital? | 4 - points |  |
| 2. Do you share the values conveyed in this hospital? | 4 - points |  |
| 3. Do you feel your work contributes to the hospital functioning? | 4 - points |  |
| **Overall job satisfaction** | **--** | -- |
| Could you please indicate your general job satisfaction on the following scale? | 10-points |  |
| **Intent to stay** | -- | -- |
| Will you keep working at the hospital within the coming year? | 4 - points |  |

^a^ For dimensions built from two items, Pearson r are provided instead of Cronbach Alphas.
