# Supplementary material for: Joint analyses of open comments and quantitative data: Added value in a job satisfaction survey of hospital professionals
Source: PLoS One. 2017 Mar 15;12(3):e0173950. doi: 10.1371/journal.pone.0173950 (PMC5352002; doi:10.1371/journal.pone.0173950)
Supplement: S2 Appendix — (DOCX) [file pone.0173950.s002.docx]

**S2 Appendix.** **Principal lexicometric characteristics of the different texts analysed.**

| Analysis steps and text analysed | Proportion of text classified (%) | Word token | Hapax | Word type | *F*_max_ (of) | TTR  (%) |
| --- | --- | --- | --- | --- | --- | --- |
| **Step 1 : Total text** | 90.2 | 76 471 | 2054 | 4766 | 4246 | 6.2 |
| **Step 2 : sub-texts** |  |  |  |  |  |  |
| Text relative to *work schedules* | 88.3 | 20 914 | 1227 | 2439 | 1202 | 11.7 |
| Text relative to *management* | 70.7 | 24 000 | 1204 | 2439 | 1238 | 10.2 |
| Text relative to *professional fulfilment* | 86.4 | 24 303 | 1352 | 2569 | 1452 | 10.6 |

Word-token, total number of words in the articles; hapax, number of words used only one time; word type, number of types of words; *F*_max_, frequency of the word most frequently mentioned; TTR, type-token ratio: ratio between the number of word types and the number of word tokens. A higher type-token ratio corresponds to richer texts.
