# Supplementary material for: Joint analyses of open comments and quantitative data: Added value in a job satisfaction survey of hospital professionals
Source: PLoS One. 2017 Mar 15;12(3):e0173950. doi: 10.1371/journal.pone.0173950 (PMC5352002; doi:10.1371/journal.pone.0173950)
Supplement: S3 Appendix — (DOCX) [file pone.0173950.s003.docx]

**S3 Appendix**. **Signed chi squars indicating significant over- and under-representativeness of independent variable modalities in each thematic class.**

|  | Work schedules | | | | Management | | | | Professional fulfilment | | |
| --- | --- | --- | --- | --- | --- | --- | --- | --- | --- | --- | --- |
| Variables modalities | Spec. sched. | Cons-traints | Qual. & safety | Abs. | Relat. instit. | Pres-sures | Comm. & clima. | Top manag. | Workload & stress | Prof. develop. | Skill recogn. |
| **Management** |  |  |  |  |  |  |  |  |  |  |  |
| Low | 3.36 | -0.96 | -0.95 | 0.00 | -8.15^**^ | 3.86 | 1.42 | -0.02 | -1.64 | 0.96 | 0.29 |
| Medium | -0.10 | 1.02 | 0.00 | -0.27 | 0.91 | -2.40 | 0.19 | 0.00 | 1.90 | -1.16 | -0.31 |
| High | -2.48 | 0.00 | 1.02 | 0.26 | 4.86^*^ | -0.33 | -3.27 | 0.01 | -0.01 | 0.01 | 0.00 |
| **Workload** |  |  |  |  |  |  |  |  |  |  |  |
| Low | 0.58 | -1.19 | -2.9 | 4.44^*^ | -2.01 | 1.43 | -1.76 | 2.75 | 9.69^**^ | -1.14 | -6.02^*^ |
| Medium | -0.18 | 0.00 | 1.85 | -1.35 | -0.06 | -3.22 | 2.84 | 0.06 | -0.14 | 2.03 | -0.71 |
| High | -0.39 | 2.75 | 0.49 | -2.49 | 6.27^*^ | 0.56 | -0.47 | -6.58^*^ | -12.17^***^ | -0.74 | 20.66^***^ |
| **Career opportunities** |  |  |  |  |  |  |  |  |  |  |  |
| Low | 6.11^*^ | -2.37 | -4.86^*^ | 1.47 | -5.92^*^ | 4.49^*^ | -0.51 | 1.44 | -3.92^*^ | 0.04 | 3.92^*^ |
| Medium | -2.50 | -0.40 | 5.13^*^ | -0.14 | 2.38 | -3.92^*^ | 0.96 | -0.48 | 2.33 | -0.22 | -1.58 |
| High | -1.29 | 6.01^*^ | 0.00 | -0.85 | 2.52 | -0.19 | -0.07 | -0.88 | 0.05 | 0.26 | -0.47 |
| **Self-fulfilment** |  |  |  |  |  |  |  |  |  |  |  |
| Low | 1.62 | -3.12 | -0.30 | 0.62 | -5.00^*^ | -0.12 | 0.01 | 6.31^*^ | 8.55^*^ | -3.21 | -2.61 |
| Medium | -1.76 | 0.59 | 5.01^*^ | -3.36 | 1.02 | 0.90 | -0.07 | -2.76 | -9.90 | 4.58^*^ | 2.41 |
| High | -0.06 | 3.06 | -4.30^*^ | 1.35 | 4.69^*^ | -0.84 | 0.01 | -2.01 | -0.01 | -0.25 | 0.29 |
| **Work-related burnout** |  |  |  |  |  |  |  |  |  |  |  |
| Low | 5.94^*^ | 2.55 | -2.29 | -5.16^*^ | 0.48 | 1.13 | 0.01 | -3.29 | -2.56 | -2.62 | 9.94^**^ |
| Medium | -4.80^*^ | 6.28^*^ | 0.48 | -0.36 | -0.50 | -0.74 | 1.10 | 0.22 | -0.89 | 2.02 | -0.05 |
| High | -0.02 | -12.77^***^ | 0.58 | 5.57^*^ | 0.02 | -0.03 | -1.83 | 1.61 | 5.61^*^ | 0.02 | -7.33^**^ |
| **Organisational commitment** |  |  |  |  |  |  |  |  |  |  |  |
| Low | -0.05 | -0.41 | -0.12 | 1.42 | -2.47 | -0.10 | -0.82 | 7.91^**^ | 1.18 | -2.31 | 0.02 |
| Medium | 2.54 | -0.76 | -0.00 | -0.84 | -0.01 | 0.34 | -0.94 | 0.29 | -0.42 | 4.24^*^ | -1.2 |
| High | -4.99^*^ | 4.55^*^ | 0.64 | -0.13 | 4.52^*^ | -0.34 | 5.36^*^ | 0.29 | -0.38 | -0.33 | 1.37 |
| **Intent to stay** |  |  |  |  |  |  |  |  |  |  |  |
| No | 4.39^*^ | -0.07 | -3.05 | 0.00 | -2.80 | 0.58 | -1.42 | 4.82^*^ | -0.13 | 0.24 | 0.00 |
| Yes | -6.13^*^ | -0.01 | 5.11^*^ | 0.02 | 4.03^*^ | -1.58 | 2.85 | -6.58^*^ | -0.01 | -0.15 | 0.18 |
| **Overall job satisfaction** |  |  |  |  |  |  |  |  |  |  |  |
| Low | 0.12 | -3.03 | 0.04 | 0.78 | -1.69 | 0.59 | -2.97 | 5.49^*^ | 3.78^*^ | -0.15 | -3.17 |
| Medium | 1.14 | 0.15 | -1.17 | -0.06 | -0.01 | -0.52 | 1.61 | -0.24 | -1.61 | 3.44 | -0.06 |
| High | -3.16 | 2.64 | 1.21 | -0.58 | 1.87 | -0.11 | 0.57 | -3.41 | -2.06 | -1.61 | 7.13^*^ |
| **Professional categories** |  |  |  |  |  |  |  |  |  |  |  |
| Physicians | 13.00^***^ | 0.39 | -10.78^***^ | -0.40 | -6.88^**^ | 9.18^**^ | -0.22 | 0.09 | 4.17^*^ | 0.11 | -6.30^*^ |
| Nurses | -15.08^***^ | -14.74^***^ | 28.76^***^ | 1.68 | 0.83 | 0.02 | -0.67 | -0.05 | 1.69 | 0.22 | -3.34 |
| Admin. | 0.08 | 3.76 | -1.37 | -0.40 | 4.45^*^ | -5.46^*^ | -0.01 | 0.03 | -3.40 | -2.94 | 12.28^***^ |
| Laboratory | 9.77^**^ | -0.52 | -10.82^***^ | 1.13 | 0.74 | 2.94 | -1.16 | -1.97 | -0.20 | -0.35 | 0.99 |
| Researchers | 0.46 | 2.10 | -0.33 | -1.75 | -1.56 | -0.96 | 4.10^*^ | 0.02 | -0.92 | 5.04^*^ | -0.84 |
| Logistic | -3.86^*^ | 10.8^***^ | 0.90 | -3.31 | -0.42 | -0.39 | -0.32 | 3.30 | -1.26 | 0.24 | 0.63 |
| Psycho-social | -0.61 | 1.65 | 0.15 | -0.52 | -3.24 | -0.54 | 5.98^*^ | 0.00 | -0.31 | -0.11 | 0.81 |
| **Facilities** |  |  |  |  |  |  |  |  |  |  |  |
| Admin. | -0.44 | 1.02 | 1.07 | -1.85 | 3.6 | -2.96 | -1.00 | 0.48 | 2.97 | -0.20 | -2.2 |
| Surgical | -5.45^*^ | -0.01 | 0.67 | 2.78 | 0.62 | 1.19 | -0.39 | -1.44 | -0.00 | 1.28 | -0.87 |
| Medicine | -0.80 | -0.24 | 1.24 | 0.01 | -0.01 | 0.87 | 0.31 | -1.86 | 5.00^*^ | -0.94 | -2.51 |
| Mixed | 0.20 | -11.43^***^ | 0.05 | 4.68^*^ | -2.05 | 2.64 | 0.82 | -0.98 | -0.25 | 6.10^*^ | -2.6 |
| Laboratory | 27.38^***^ | 2.60 | -17.87^***^ | -4.68^*^ | -0.56 | 0.62 | -0.78 | 0.84 | -0.00 | -1.75 | 1.46 |
| Logistic | -3.02 | 13.86^***^ | -0.33 | -0.44 | -0.89 | -0.40 | -2.75 | 10.50^***^ | -0.67 | 0.09 | 0.39 |
| Psychiatry | 0.14 | 1.10 | 0.11 | -2.89 | -0.13 | -1.70 | 0.67 | 0.60 | -5.70^*^ | -0.50 | 10.32^**^ |
| Research | -0.15 | 0.10 | 0.21 | -0.16 | 6.83^**^ | -6.69 | 0.43 | -0.78 | 0.16 | -1.92 | 0.6 |
| Hierarchical level |  |  |  |  |  |  |  |  |  |  |  |
| Managers | -2.20 | 0.87 | 0.05 | 0.30 | -5.18^*^ | 0.09 | -0.39 | 7.15^**^ | 2.49 | 0.46 | -5.31^*^ |
| Employees | 2.20 | -0.87 | -0.05 | -0.30 | 5.18^*^ | -0.09 | 0.39 | -7.15^**^ | -2.49 | -0.46 | 5.31^*^ |

All chi squares have 1 df; * p<0.05; **p<0.01; ***p<0.001. Spec. sched., special schedules and rest; constraints, professional versus private constraints; abs., absences and replacements; qual. & safety, healthcare quality and patient safety; relat. instit., relationship with the institution; comm. & clima., communication and work climate; pressures, pressures of the hierarchy; Top manag., top management; workload & stress, workload and stress; prof. develop., professional development; skill recogn., skill recognition.
